# Advanced Materials for Oral Application (Volume 2)

**DOI:** 10.3390/ma18051042

**Published:** 2025-02-26

**Authors:** Laura-Cristina Rusu, Lavinia Cosmina Ardelean

**Affiliations:** 1 University Clinic of Oral Pathology, Multidisciplinary Center for Research, Evaluation, Diagnosis and Therapies in Oral Medicine, “Victor Babes” University of Medicine and Pharmacy, 300041 Timisoara, Romania; laura.rusu@umft.ro; 2 Academic Department of Technology of Materials and Devices in Dental Medicine, Multidisciplinary Center for Research, Evaluation, Diagnosis and Therapies in Oral Medicine, “Victor Babes” University of Medicine and Pharmacy, 300041 Timisoara, Romania

This editorial aims to present the contributions published in the second volume of the Special Issue “Advanced Materials for Oral Application”, journal *Materials.* Volume 2 aimed to focus on the recent advances in this attractive field of research, encouraging a multidisciplinary approach to the subject.

Compared to the first volume, which consisted of fourteen papers, the second volume gathered twenty-six valuable manuscripts: twenty-four original research articles and two review papers, authored by leading scientists and scholars across the world with expertise in materials for dental application. These articles explore a wide variety of dental materials for direct and indirect restorations, for endodontic use, and for dental implants, as well as their properties and interactions with the oral environment. Three-dimensional bioprinting in dentistry was also assessed, and two new dentifrices were presented.

Prevention in dentistry is of the utmost importance, as it helps in maintaining good oral health. Novel formulations of more effective dentifrices are constantly being attempted. The study by Livne et al. aimed to test the effect of adding a mucoadhesive agent, namely hydroxyethyl cellulose, to an herbal extract solution containing lavender, echinacea, sage, and mastic gum, attempting to increase its bioavailability and efficacy. This formulation provides a platform for the future development of a caries-preventing toothpaste, aiming to reducing biofilm formation and the virulence of the cariogenic bacterium Streptococcus mutans [1]. Florea et al. attempted to develop functional toothpastes with combined enamel remineralization and antibacterial effects using nano-hydroxyapatites and birch extract, and they obtained the most suitable formulation out of eleven choices [2].

Materials used in restorative dentistry are being also subjected to continuous improvement in term of physical properties, aesthetic appearance, and durability, with direct composite materials being the first choice for most patients. The essential factor in providing a long-lasting restoration is the marginal seal. Restoring cervical lesions with composite resins has always been a challenge, as stated by Sengar et al. Their study aimed to evaluate and compare the microleakage of a flowable composite resin using an etch and rinse adhesive system, a self-etch adhesive system, and a self-adhesive flowable composite resin in Class V cavities. Despite the fact that none of the of the tested adhesive systems were free from microleakage, the etch and rinse adhesive showed the lowest microleakage values [3]. Different irradiation times and light intensities have been proven to affect the mechanical properties of direct composite restorations. Their successful polymerization can be evaluated by their hardness; the study of Maximov et al. aimed to investigate the influence of different photopolymerization parameters, such as light intensity, irradiation time, and layer thickness, on the microhardness of three types of dental composites (conventional, bulk-fill, and flowable). The study has shown that the significance of the factors influencing the microhardness is defined mainly by the composite resin composition, which is similar for the conventional and flowable composites, but different for the bulk-fill [4]. Another study involving direct composite resins was authored by Klimek et al. who aimed to compare the properties of a commercial hybrid composite with experimental composites doped with different amounts of hydroxyapatite. After determining the hardness, static strength properties (compression and bending), dynamic properties (impact strength and fracture toughness), and wear resistance, they concluded that the hydroxyapatite content had a great impact on the mechanical properties of the composite resin, with the best results being obtained when adding up to 5% wt [5]. The in-vitro study by Aldowsari et al. aimed to assess the shear bond strength of a new self-adhesive restorative material to the dentin of extracted permanent teeth previously treated with 38% silver diamine fluoride and to compare it with a resin-based composite and a resin-modified glass ionomer cement. The best results were obtained when using the resin-based composite, but the shear bond strength of the new self-adhesive restorative material was within the clinical acceptable limits, making it suitable for masking the discoloration caused by silver diamine fluoride application [6]. Microbial adhesion on dental restorative materials may jeopardize the long-term outcomes of restorative treatment. The goal of the in vitro study of De Angelis et al. was to assess the capability of *Candida albicans* to adhere to and form biofilms on the surfaces of dental composites with different formulations which were subjected to same surface treatments and polymerization protocols. Based on the results, the conclusion was that different formulations of commercially available composite resins may interact differently with *C. albicans* [7].

Dentinal hypersensitivity is a painful condition which may affect patients of any age, and is usually associated with exposed dentinal surfaces. Due to its negative effects on psychological and emotional wellbeing, there is continuous interest in finding new and efficient means of treating it. The randomized controlled trial of Rai et al. aimed to evaluate the effectiveness of a single chair-side application of NovaMin^®^, a bioactive glass containing calcium sodium phosphosilicate, in reducing dentin hypersensitivity following ultrasonic scaling. The study, carried out over a one-month period, concluded that NovaMin^®^ is efficient in providing rapid relief from dentinal hypersensitivity [8]. Sahin et al. evaluated the effects of a novel nanohydroxyapatite gel and Er: YAG laser on the surface roughness, surface morphology, and elemental content after dentin hypersensitivity treatment, and concluded that these treatment methods could provide promising results in terms of tubular occlusion efficiency. Laser treatment resulted in significantly lower surface roughness, which could help in preventing dental plaque accumulation [9].

One dental field which has undergone great development within the recent time period is endodontics. The ultimate goal of endodontic treatment is to obtain a three-dimensional, tight canal seal. To achieve a good three-dimensional seal, the removal of the smear layer becomes mandatory, as stated by Rajamanickam et al. Their study aimed to assess the difference in debris accumulation and smear layer formation while using automated root canal irrigation and conventional syringe needle irrigation, and they observed no significant difference between the results of the two irrigation techniques [10]. The quality of the three-dimensional seal highly depends on the materials used. Over the years, various endodontic sealers have been developed. Hydraulic calcium silicate-based sealers significantly changed the fundamental principles of root canal fillings due to the lack of shrinkage and long-term dimensional stability. However, their drawback resides in the difficulty of removing them from the root canal system, even mechanically, in case of retreatment. Drukteinis et al. tested the ability of citric acid to dissolve hydraulic calcium silicate-based sealers and demonstrated its efficacy with no adverse effects on root canal dentine [11]. Characterized by high biocompatibility, low cytotoxicity, and viscosity, calcium silicate-based sealers have been considered to improve canal filling quality. The aim of the study of Ashi et al. was to investigate the influence of the mechanical properties of three different calcium-silicate-based cements on the stress distribution of three different retrograde cavity preparations. Their conclusions were that the apical preparation design influences stress distribution and that stiffer cements offer an optimal retrograde treatment with less stress in the root [12]. Following endodontic treatment, the teeth need reconstruction, which is carried out by using different post systems made of different materials, with different characteristics. Treatment failure in teeth restored with endodontic posts is frequently due to the loss of bond between the post and the tooth structure. Push-out strength tests, used by Habib et al. in their study, are widely accepted to evaluate adhesive strength. They aimed to assess the push-out strength of different sizes of fiber and metal posts, which were luted with a dual-cure resin-based cement to natural dentin. They also analyzed the type of failure and concluded that fiber and metal posts showed almost similar values of bond strength, while increasing the size of the post resulted in better retention due to the wider bonding surface area. However, the post size should be carefully chosen to avoid weakening of the root structure. Adhesive failure was the most prevalent type [13]. Endocrowns had been introduced as a more conservative approach to rehabilitate endodontically treated teeth with significant losses in coronal structure, avoiding further weakening of the root. Endocrowns are associated with lower stress concentration and higher fracture strength compared to post-supported conventional crowns. Darwel et al. aimed to assess the stress distribution in molars restored by CAD/CAM manufactured endocrowns from translucent zirconia, as well as to evaluate the biomechanical behavior of translucent zirconia endocrowns compared to endocrowns made of zirconia-reinforced lithium silicate ceramic, lithium disilicate glass ceramic, polymer-infiltrated ceramic, and resin nanoceramic. Their conclusion was that resin nanoceramic caused high stress concentrations and displacement in dental structures, thus being a less suitable material for endocrowns. Translucent zirconia was shown to be the best for endocrowns, as it absorbed stresses and showed low displacement within them as well as in the dental tissues [14].

Dental implants represent one of the greatest advances in oral rehabilitation. Titanium implants are considered the most common type of implant because of their durability and functionality. However, aesthetic failure of implant rehabilitation is quite frequent, being related to the specific color of the titanium screws. This is the reason why several technologies have been developed for the production of colored titanium surfaces, including laser. Zara et al. aimed to investigate the relationship between novel laser-colored surfaces and peri-implant soft tissues and confirmed the innovative physical titanium improvements due to laser treatment, which is biocompatible and allows a wide color palette to be obtained while maintaining the roughness of the titanium surface and being able to promote the growth of soft tissues [15]. Immediate implant placement eliminates the need for multiple surgeries and reduces treatment time. Thus, the study of Comuzzi et al. aimed to compare the stability of two conical implants and a cylindrical one inserted into low-density polyurethane blocks with or without a cortical lamina, which potentially mimicked the post-extraction condition. They concluded that the conical implant shape could be considered the best-performing one in artificial post-extraction conditions due to the higher primary stability values [16]. The micro- and nanostructures and wettability of titanium implant surfaces are essential for osseointegration. Improved cell response and shorter healing time are the results of combining hydrophilicity and nanostructure. The study of Illing et al. aimed to investigate the biological response to different wettability levels and nanostructure modifications in titanium surfaces. The additional nanostructures created by plasma etching with fluorine gas demonstrated improved fibroblast cell viability, but did not lead to improved early osseointegration. They concluded that other factors besides surface roughness and wettability play decisive roles in cell attachment, cell viability, and osseointegration [17].

The main goal of dental treatment concerns either the regeneration of diseased tissues or their replacement with prosthesis. Three-dimensional bioprinting uses bioinks to fabricate complex organ structures and functional tissues that can support live cells and other biological factors. By means of 3D bioprinting, tailored tissue-engineered constructs with customized complex architecture and properties can be speedily manufactured [18]. The scoping review of Mohd et al. aimed to explore the 3D bioprinting technologies, biomaterials, and cells used for dental applications. According to the authors, 3D bioprinting has shown promising results for periodontal ligament, dentin, dental pulp, and bone regeneration applications, but, regrettably, is not yet close to being a clinical reality [19].

Edentulism is a worldwide phenomenon with both esthetic and functional repercussions, being considered a reflection of the patient’s history of dental conditions and treatments. The field of prosthetic rehabilitation has changed drastically as a result of advancements in materials and digital technology, aiming to preserving tooth structure and offering the best treatment option. Adhesive techniques need less invasive tooth preparation. Three different designs (one fixed-fixed and two cantilever designs) of resin-bonded bridges were compared and evaluated by Narwani et al. according to tensile bond strength. All designs used both the Rochette and Maryland types of retainers. The fixed–fixed design proved superior, while Maryland bridges showed higher bond strengths across all framework designs, implying that they may demonstrate lower clinical failure than cantilever designs and Rochette bridges [20].

Among the most recent and performant technologies used in prosthetic dentistry, computer-aided design and computer-aided manufacturing (CAD/CAM) enables subtractive or additive fabrication of various types of dental prostheses and appliances. Addugala et al. analyzed the marginal discrepancy and internal adaptation of copings fabricated using three types of resin patterns with subtractive (milling) and additive technology (3D printing), namely, milled polymethyl methacrylate resin and digital light-processed acrylonitrile–butadiene–styrene and polylactic acid patterns. The patterns were subsequently casted with a Co-Cr alloy, and the internal and marginal gaps of the copings were microscopically observed. Copings fabricated from the milled PMMA group had the best marginal fit, while copings fabricated with the PLA 3D printed group had the best internal fit [21].

Recent advances in CAD/CAM technologies have allowed for the manufacturing of different types of materials for the CAD/CAM milling process: metal-based, ceramic-based, and resin-based.

Biofilm formation on hybrid resin-based CAD/CAM materials was evaluated by Tzimas et al. in a comprehensive review. As biofilms play an important role in restoration failure, by facilitating secondary caries appearance at the restoration’s margins and by altering the restorative material’s surface characteristics, the current literature describes a possible interaction between biofilm formation and the surface of the restorative material [22].

Zirconia-based ceramic CAD/CAM blocks are a frequent choice because of their versatility, combining high strength with improved esthetics. Their lack of translucency has been overcome by the latest generations of Y-TZP zirconia, with improved properties and wider indications for both monolithic and veneered restorations. The interest for zirconia-based ceramics is highlighted by six articles published in this Special Issue, assessing this type of block [14,23,24,25,26,27]. Chen et al. investigated the effect of restoration thickness on the fracture resistance of translucent 5-YZP crowns cemented with different cements and subjected to different surface treatments compared to 3-YZP crowns, with detailed results available in the manuscript [23]. Sokolowski et al. investigated the shear bond strength of resin-based luting cement to zirconia ceramics after different surface treatments, aiming to determine the effect of a new etching technique on zirconia and assessing the effect of using primers on the bond strength of zirconia luted with resin cement. Their study concluded that etching of zirconia caused changes in its surface structure and a significant increase in the shear bond strength between zirconia and the resin cement. The use of primers positively affected the adhesion between the resin cement and zirconia [24]. The study of Nasarudin et al. explored the biocompatibility of 3-YZP on three-dimensional oral mucosal models constructed using human gingival fibroblasts and an immortalized human oral keratinocyte cell line co-cultured on an acellular dermal matrix. The study proved the excellent biocompatibility of 3-YZP, indicating its high potential for clinical application as a restorative material [25]. The in vitro study of Rosentritt et al. aimed to compare the cutting efficiency of diamond grinders on zirconia and resin-based composite materials by weight measurements of the material before and after grinding. The grinders showed significantly different initial wear removal and durability. With grinding on zirconia, it took five times as long to remove comparable weight [26]. Smielak et al. aimed to determine whether sandblasting was accompanied by the phenomenon of driving abrasive particles into the conditioned material. Their study assessed disks made of chromium/cobalt, chromium/nickel, titanium, and sintered zirconium dioxide, sandblasted with three different grain sizes of aluminum oxide at three different pressures. After sandblasting, abrasive particles were found on the surfaces of the materials, with the amount being dependent on the hardness of the processed material. Increased grain size and pressure led to an increased amount of embedded grain [27].

In summary, this Special Issue of *Materials*, titled “Advanced Materials for Oral Application-Volume 2”, represents a valuable collection of twenty-six cutting-edge research and extensive review articles from across the world, demonstrating the great potential of novel, durable, and high aesthetic dental materials and informing the readers of the current challenges and future directions in this domain.

The Guest Editors would like to thank all contributing authors for the success of the Special Issue. This Special Issue would not have been of such quality without the constructive criticism of the Reviewers. Special thanks and appreciation go to the MDPI *Materials* Section Managing Editor for her most valuable support and collaboration.
